# Supplementary material for: Catastrophic health care spending in managing type 2 diabetes before and during the COVID-19 pandemic in Tanzania
Source: PLOS Glob Public Health. 2023 Aug 22;3(8):e0002180. doi: 10.1371/journal.pgph.0002180 (PMC10443863; doi:10.1371/journal.pgph.0002180)
Supplement: S1 Table — (DOCX) [file pgph.0002180.s001.docx]

**Supplementary materials**

**S1 Table: Distribution of zero payments**

|  | **Pre-COVID-19** | | | **During COVID-19** | | |
| --- | --- | --- | --- | --- | --- | --- |
|  | **Worse-off** | **Better-off** | **Total** | **Worse-off** | **Better-off** | **Total** |
| **Share of zero payments** |  |  |  |  |  |  |
| Total cost | 4.8% | 7.2% | 6% | 7.5% | 7.6% | 7.6% |
| Healthcare cost | 45.8% | 51.6% | 48.7% | 53.7% | 52.5% | 53.1% |
| Transport cost | 8.8% | 9.9% | 9.3% | 10.1% | 11.2% | 10.7% |
|  |  |  |  |  |  |  |
| **Observation (n)** | 227 | 223 | 450 | 227 | 223 | 450 |
