# Supplementary material for: Catastrophic health care spending in managing type 2 diabetes before and during the COVID-19 pandemic in Tanzania
Source: PLOS Glob Public Health. 2023 Aug 22;3(8):e0002180. doi: 10.1371/journal.pgph.0002180 (PMC10443863; doi:10.1371/journal.pgph.0002180)
Supplement: Attachment — Submitted filename: PLOS GPH_Response sheet.docx [file pgph.0002180.s002.docx]

**Reviewer(s)' Comments to Author: Manuscript Number: PGPH-D-23-00508**

Please use the space provided to explain your answers to the questions above. You may also include additional comments for the author, including concerns about dual publication, research ethics, or publication ethics. (Please upload your review as an attachment if it exceeds 20,000 characters)

**Response:**

Reviewer #1:

1. A timely manuscript presenting key findings based on primary data among a critical subgroup of the population. The study design (pre-post retrospective survey) implies a certain risk of recall bias, thus it is understandable that household expenditure was not collected and instead income (which tends to be less varying) was assessed.

**Response: We agree with the reviewer about the risk of recall bias since we collected data retrospectively, and the concern of using a less varying measure of living standard (i.e., income). All these concerns (recall bias and the use of income) have been discussed among study limitations, indicating that we are aware of those issues.**

2. Some pleasant observations in the manuscript include i) that the analysis appears to be consistent with the study objectives, and is easy to follow along. There's evidence of rigorous behind the analysis, for instance there's demonstrated understanding of the causes of changes in healthcare costs (e.g increase in proportion of patients conducting home based testing / monitoring of blood sugars post COVID) and ii) that the results are presented in easy to interpret format, showing both absolute and relative differences for instance in Table 3.

**Response: We really appreciate the reviewer for acknowledging our effort to align all sections and text for a reader to follow.**

3. I think it may be useful, if data are available, to understand the socioeconomic distribution of those that reported no payments for health care. One limitation with the calculation of risk of catastrophic payments is that it considers all participants surveyed as a population at risk, knowing that there are those who report zero values in the numerator because they did not access health services and therefore had no opportunity to incur costs. A disaggregation of the income of those who report zero numerator values (zero healthcare costs) may support a hypothesis of increased financial barriers to access (especially post COVID).

**Response: We have analysed the distribution of zero payments by income, and added as an appendix table. First, almost 50% of patients did not pay for health care over time; second, these shares increased slightly during COVID-19 especially among the poor. This text has been added in the result section and partly in the discussion.**

**It is typical for incidence of catastrophic to use all surveyed individuals/ households in the denominators. It is not problematic because those with zero payments are not included in the numerator. It is only those who spend at least 10% (depending on a threshold used) of their income for healthcare payments. If zero payments are removed in the denominator, the incidence of catastrophic spending will be overestimated (because it will show a catastrophic incidence among those who incurred healthcare cost only). In fact, this will report a higher incidence of catastrophic in settings with low rate of paying out-of-pocket, which may lead to a misleading conclusion. For instance, in a survey covering 100 people, 20 people paid OOP, 10 out of 20 paid above 10%-threshold of their income: catastrophic incidence equals (10/100=10%), but removing people with zero payments will give an incidence of (10/20=50%).**

4. Finally, please mention if the changes in costs pre and post COVID are adjusted for inflation of local currency over the period, or otherwise quote the average inflation between the two time points.

**Response:**

5. Thanks again to the authors for a very relevant piece of work!

**Response: Thank you!**

**Reviewer #2:**

GENERAL COMMENTS
The authors of this manuscript provided a information on catastrophic health care spending among patients with Type2 Diabetes in the pre and post COVID-19 era in Tanzania. The study highlighted the potentials of the various factors, and their contributions towards incurring catastrophic health expenditure among the Type2 Diabetes patients in their country. Their findings contribute to further understanding of the complexity of health care financing in their country as it affects Type 2 diabetes patients and therefore relevant.

**Response: Thank you!**

TITLE
This adequately captures the study

**Response: Thank you!**

ABSTRACT
This adequately described their study and concise.

**Response: Thank you!**

INTRODUCTION
This is generally adequate and provided the background for the study.

**Response: Thank you!**

METHODOLOGY
This is generally adequate and well presented. However, the authors could improve the flow of this aspect by explaining how they classified the participants into the two income groups namely: poor and non-poor. Which instrument did they use? Providing this information is very important for reproducibility.

**Response: We have expanded the description on how we generated income subgroups. We basically used a principal component analysis based on 42 items of household characteristics and asset ownership to generate a wealth score for each household. Then, we ranked all 450 households according to the wealth index/ score and categorised into two equal sized subgroups (the better-off and worse-off). But, for simplicity in interpretation, this paper referred the two subgroups as non-poor and poor.**

DISCUSSION
Generally, well presented and discussed.
However, the authors merely duplicated their results in the first paragraph of this section without comparing them to published literature on the subject. The authors would do well to address this concern. They need to relate these results to published literature or they take off this paragraph as it sounds like a duplication if no comparison is made to published literature.

**Response: The first paragraph in the discussion has been removed to avoid duplication of statements.**
