# Supplementary material for: Catastrophic health care spending in managing type 2 diabetes before and during the COVID-19 pandemic in Tanzania
Source: PLOS Glob Public Health. 2023 Aug 22;3(8):e0002180. doi: 10.1371/journal.pgph.0002180 (PMC10443863; doi:10.1371/journal.pgph.0002180)
Supplement: Attachment — Submitted filename: PLOS GPH_Response sheet2.docx [file pgph.0002180.s003.docx]

**PGPH-D-23-00508R1
Catastrophic health care spending in managing type 2 diabetes before and during COVID-19 pandemic in Tanzania**

PLOS Global Public Health

Reviewers’ comments
==============================

1. Please be consistent with abbreviations- sometimes T2D is used whereas type2 diabetes is spelled out elsewhere.

**Response: Thanks for that observation. We have ensured consistency in using the abbreviations. However, it is important to note that other sentences may refer to diabetes in general (which includes T1D and T2D).**

2. Carefully proof-read for typos

**Response: We have read the whole manuscript and asked an English speaker to proof-read as well.**

3. Regarding classification of income groups into "poor/non-poor", i think ascribing poverty without justification may be inapropriate. Consider using a more justifiable terminology for the classification.

**Response: We have edited the manuscript accordingly. We decided to use two income groups – the better-off and worse-off, as opposed to poor vs. non-poor which requires the use of poverty measures.**
